# Extra Supplement.—The Nursing Mirror

**Published:** 1888-10-20

**Authors:** 


**The Nursing Mirror.**

All Contributions for this Supplement should be addressed "The Nursing Mirror," care of The Editor, 38, Old Jewry, E.C.

### En Passant.

IN THE NURSE'S ABSENCE.—Last week, Hannah Clifford, wife of a Nottingham upholsterer, died from shocking injuries received by throwing herself from a window. It seems that for some months deceased's mind had been unhinged, and she had been attended by a nurse; in the temporary absence of the latter, Mrs. Clifford opened the bed-room window and threw herself out, falling a distance of forty feet.

EMIGRATION.—Lady Samuel, of 15, Courtfield Gardens, to whom applications should be made, is making arrangements for despatching some English nurses to Sydney. On their arrival in Australia, the nurses are taken into a home, where they receive five shillings a-week. When out nursing they receive twenty shillings, or, if the case be an infectious one, thirty shillings. Washing and uniform are found for them, and work is guaranteed. Before leaving England nurses sign an agreement to serve the home for one year, and they must have a doctor's certificate as to their state of health. They pay their own passage money out, but if unable to do so, the money is advanced here and deducted, £5 quarterly, from their salaries in Sydney.

NURSING AT NETLEY.—Last week, in our invaluable contemporary the *Queen*, there was an interesting article on the Netley Hospital, which gave a good deal of information about Army Sisters. Mrs. Deeble, the widow of an officer, is the superior and founder of this noble band of workers, and she always prefers to take as nurses the daughters of officers, because they are familiar with the routine of army life. The nurses serve the usual three years' training in some civil hospital, and then go to Netley for six months on probation. If they are suitable and like the life, they are then taken on as sisters at a good salary. The work is chiefly of superintendence, the actual nursing being done almost entirely by the orderlies. There are at present about sixty Army Sisters, ten or twelve of whom are at Netley.

AN AMERICAN WOMAN'S WORK.—"Once a nurse, always a nurse," is not true of many of our sisters over the water. There, where there are so many more openings for women, a nurse when getting too old or too weak for her work, can turn her energies to other things. Theresa Kelly, who is now superintendent of a bookbindery in Ohio, entered a hospital during the war as a nurse. Her services were so valuable she was sent to the front, and received a commission as an orderly sergeant from the 5th Ohio Infantry. What a pity it is that though military nurses often received marks of honour, there is no decoration ever given to nurses in civil hospitals, where the work and the danger are equally great.

THE PENSION FUND.—The meeting at St. Thomas's on October 10th, to hear the new manager of the Pension Fund expatiate on its progress, was both interesting and amusing. Dr. Potter had evidently lately visited the Irish Exhibition and kissed the blarney stone, but his speech seemed more popular than the severely-financial and business-like statements of Mr. Clifford and Mr. Burdett. Perhaps this was because the audience were mostly women. There were a great number of nurses present, the provinces being specially well represented; but, unluckily, the nurses and matrons of some of our largest hospitals were unable to attend. With the best will in the world a hospital nurse cannot get leave when she would. At the "London," for instance, it was lecture night, so not a single nurse could get to St. Thomas's. The chief news told at the meeting was that the fund was fairly started, over £3,000 having already been paid in premiums by nurses.

A SPECIMEN OF PROVINCIAL NEWS.—The following extraordinary statement is taken from the *Melton Mowbray Times*, "Mr. R. D. Roberts, whose name is well known in connexion with the extension of university teaching among the miners of Northumberland, is about to marry Miss King, the matron of St. Bartholomew's Hospital, in London. He is a firm believer in the equality of the sexes, and does not intend that marriage shall cut short his wife's professional career, as is generally the case. It has accordingly been agreed that he shall continue to lecture, and she to nurse, just the same as heretofore. The experiment will be watched with a good deal of interest." We were under the belief that Miss Stewart was matron of Barts.

NEWS FROM TRURO.—The annual general meeting has taken place at the Royal Cornwall Infirmary, and the governors were able to inspect the new cubicles for nurses, made out of an old ward on the lower floor. Each nurse now has a separate sleeping apartment, and a common sitting room has also been comfortably furnished. This hospital makes large advances every year, both in the number of its patients, and in improvements. At the harvest festival held on October 4th, the collection, amounting to £1 5*s.*, was devoted to the foundation of a samaritan fund. The services were very hearty, and the chapel beautifully decorated. Under the able superintendence of Miss Burgess, this is now a thorough and comfortable training school for nurses.

DEATH FROM MISADVENTURE.—Such was the merciful verdict at the inquest on the youth Jeffers, who was accidentally poisoned at Belfast by a nurse. How the sad fatality occurred it seems impossible to imagine, for we learn from the evidence that the bottle was marked—"Carbolic Acid, Crude Poison." Another death from misadventure has occurred lately at the Hardwich Hospital, Dublin, through the mistake of a student; and a promising young soldier was last week poisoned in France by the mistake of a hospital orderly. Now there is a very serious lesson to be learnt from all these cases, and on probationers especially would we impress the necessity of always reading the label before pouring out a medicine. It is a mere matter of habit in which all nurses should train themselves, to read the label of every bottle every time it is used. No matter how constantly the same medicine has been given from the same bottle, absolute security from mistake can only be attained by learning the habit of always looking at the label. Remember this, nurses, for repentance is vain once a fatal mistake is made. Here is the story of the French victim; imagine the feelings of the poor orderly at whose door his death lies. "The second son of Baron de Carayon-Latour was quartered with his regiment at Meaux, and, feeling ill during the manœuvres at Châlons, he was put on the sick-list, and obtained leave to go home to his family. Before leaving camp he went to the hospital for some medicine, and received from one of the soldier-assistants a packet of white powder, which he dissolved and swallowed. M. de Carayon-Latour then entered the train for Paris, and during the journey the poison took effect, and caused him terrible convulsions. On arriving at home the young man immediately went to bed, telling his father that he was poisoned. The Baron, almost beside himself with grief, sent for Doctor Brouardel and two other medical experts, but when they arrived they found that the case of the patient was hopeless. The young man died, asking his father not to have the hospital-assistant punished, as he had given the poison, which was probably arsenic, by mistake. M. de Carayon-Latour was only twenty-two years old, and was serving a term of five years in the army."

### Original Articles.

### A MATRON'S DUTIES.

At the request of some of our readers we are going to try and follow a nurse's progress a step higher, and consider what may await her when she attains to a matronship. The subject is fraught with difficulties, because the duties of no two matrons are exactly similar, and what may be demanded of one may be denied to another. To take the duties of a matron in a cottage hospital first: they include the charge of the linen, stores, and drugs, the buying and keeping in order of the first two, and the keeping of a correct list of all three. Not unusually the matron is required to have a slight knowledge of dispensing, so that in cases of emergency she can make up medicines. Urine testing and dressings, and many of the minor duties of a house surgeon, are also often left to the cottage matron. From the picture given in Miss Lonsdale's *Life of Sister Dora*, one might almost imagine her to have been the supreme ruler over the little Walsall Hospital, but such an amount of responsibility and surgical work are the exception. Where there is no doctor on the premises, though, and accidents are brought straight to the hospital, it can be imagined that a nurse might properly overstep the limits which tie her in larger buildings, and profitably perform duties there left to the house surgeon. The matron has also the supervision of patients, the giving of medicine, the serving of meals, and often takes an active part at operations. Here her duties are coincident with those of a Sister in a larger establishment, and she would do well to regulate her hours according to their usual time-table. She also has to attend to the out-patients. The servants—who usually consist of one woman to cook and scrub, and a porter who combines the character of gardener—are also under the matron's charge, and she must receive all visitors who wish to inspect the hospital. Even in the smallest hospitals it is now usual to train at least one probationer, as with her help a certificated nurse can manage twenty beds. The training must be done by the matron, who usually does the dressings, &c., herself the first few days, while the probationer stands by and hands what is required, clears away soiled dressings, and learns all she can. The matron may find it necessary to sometimes undertake the night duties, when her nurses are hard pressed, and at all times there should be a bell in her bed-room which can be rung from the wards, so that in case of emergency she can be immediately aroused. She is so entirely in charge of the building and its inmates and contents, that her position is more like the head of a family.

Turning to the duties of a matron in a small infirmary containing perhaps forty or more beds, and having a resident house surgeon, we cannot do better than to print the following rules of just such an institution :—

I. The matron shall be in charge of the domestic arrangements in the infirmary, and be responsible to the managers, but more immediately to the House Committee, for the good order and government of the household.

II. She shall visit all the wards and the house generally, and inspect the pantry, kitchen, store-room, servants' rooms, &c., at least once a-day, and shall carefully examine the state of the wards, and see that the nurses are attentive to their duties, and that the patients conform strictly to the regulations.

III. She shall take care of the provisions, coals, and stores, and see that sufficient supplies are provided and kept up, and, when goods are required, she shall write out the orders to be submitted to the House Committee by the clerk. She shall check the quantities and qualities of the various articles as they are received, and see that they correspond with the contracts, certifying the same to the clerk.

IV. She shall issue all supplies, as required by the Dietary Store Sheet, as daily sent to her for the different meals.

V. She shall be careful that all the provisions are good, clean, and wholesome. She shall be present in the kitchen daily to see that they are properly cooked and served (for the patients), according to the Kitchen Diet Sheet furnished daily by the house surgeon.

VI. She shall, subject to the approval of the convener of the House Committee, hire, suspend, and dismiss the nurses and female servants. She shall keep the time and pay books of all the nurses and female servants of the infirmary, and shall give them to the clerk for transmission to the treasurer, who will pay the amounts to the respective parties as may be arranged.

VII. In the months of January and July she shall make an inventory of all the furniture, blankets, bedding, napery, &c., then in use in the stores, which she shall submit to the House Committee, who, having satisfied themselves as to its correctness, shall report thereon to the managers at their next meeting.

VIII. She shall supervise and instruct the nurses and probationers in all branches of nursing. She shall assist the nurses, when necessary, in the performance of their duties.

IX. She shall instruct the senior nurse for the time being in the general duties of the management of the household, to an extent sufficient to enable her to undertake charge of the domestic arrangements when required.

X. In the temporary absence of the matron, the senior nurse shall assume domestic charge of the household. The matron shall not leave the infirmary without intimating her intended absence to the senior nurse.

XI. She shall receive from the house surgeon instructions, when necessary, regarding the nursing and treatment of any case, so that in supervising the nurses she may satisfy herself that they are performing their duties carefully and efficiently.

XII. For small and emergent orders she shall keep pass-books with all the tradespeople, giving written reasons for such orders, initialing as a receipt each article supplied, and submitting the books through the clerk to the inspection of the House Committee.

XIII. She shall furnish the clerk weekly, on Wednesdays, with a list of all articles of food, liquor, &c., she may have required for the use of the household, apart from the patients, during the previous week.

XIV. She shall retain charge of the wines, spirits, and beer, and issue them to the patients in accordance with the list compiled by the house surgeon from the ward wine books

XV. She shall take charge of such money or articles of value as patients may have in their possession when admitted.

This certainly seems a formidable list of duties, and one may well say that a matron must needs be a Jack-of-all-trades. Then in some hospitals the matron has charge of all the housekeeping and the supervision of the nurses' rooms, &c., but is allowed no share in the nursing whatever. She is not allowed to be present at operations, and one matron, who in passing round the wards saw a nurse doing a faulty dressing and corrected her, was herself corrected by the committee for going beyond her province. This is why the post of matron has its disadvantanges—because there is often little or no nursing connected with it, but merely housekeeping and general management. It is not the good nurse who usually makes the good matron; it is only a good manager, a keen, businesslike woman, one with a natural aptitude for organising, who is successful as a matron. Then, in the yet larger hospitals it is necessary that a matron should have a good presence, a pleasant manner, and a general *savoir faire*, that she may deal properly with the numerous visitors and others with whom she is thrown in contact. In the biggest London hospitals it takes the matron three hours to walk through all the wards, speaking a few words to each Sister, and giving a careful glance into all corners. She has also to receive some twenty heads of departments every morning, to hear their reports; this takes at least an hour and a-half; and then her correspondence is enormous. Very little of the housekeeping, however, falls to such a matron's share—she generally has an assistant to whom that branch is relegated, so that the whole of her time and intelligence can be given to maintaining her position as head of the nursing staff, over whom she reigns supreme. The whole social tone of a hospital is usually taken from the matron, and if you visit a building where the porter receives you curtly, the nurses ignore you, and the patients stare rudely at you, you may be sure that the matron herself falls short of the proper standard. If the porter directs you courteously, the nurses come smiling forward to know your wishes, and the patients are sleeping, reading, or chatting, you may be sure that the matron is a splendid specimen of womanhood. Few, very few, are fit to fill these important posts; the perfect matron is not one in a thousand, but one in a million.

### Everybody's Opinion.

*[Correspondence on all subjects is invited. No communications can be entertained if the name and address of the correspondent is not given, or unless one side of the paper only be written on.]*

### THE BRITISH NURSES' ASSOCIATION.

### ONE VIEW.

We publish the following correspondence by request; the names are withheld, for obvious reasons:

Dear Matron,—I still feel a bit of an exile at my out-of-the-way post, and often long to be back at dear busy ——. I joined the British Nurses' Association, hoping to get some good, or at least some feeling of fellowship out of it; but, except a card signed "Helena," and notice of two members' meetings, I have got nothing for my half-crown. I should like your advice before paying my subscription again, for I cannot afford to waste my scanty wages if there are no real advantages to be gained.

Dear Nurse,—I am glad of your letter just received, for I myself have been puzzling over the question of the British Nurses' Association. The only good you are ever likely to get may be the registration of nurses at some far-off date—if the association and you both manage to exist for a great number of years. And what benefit it will be to you who hold our certificate, to be on a register with all sorts and conditions of nurses, I do not know. The present plan of the association is to register all women who have been engaged in nursing three years. Perhaps you say you would much prefer not to be classed amongst the rabble. But if it is made a matter of law, you will not be able to pursue your vocation unless you are on the register. Why not pay your half-crown to the Pension Fund, which will then be ready to aid you if you are ill?

Dear Matron,—I think I follow your argument that the British Nurses' Association is of little use to me; but looking to the future of my profession, and thinking of others, will it not do good work? To be able to pass a public examination, and be registered, even as a physician is, as a fit person to practise, will surely be useful to nurses, and to the public, in the future? Then the *Lancet* has said such a lot against the Pension Fund, I shall not join it till it is started. Nurses are too poor to run risks.

"Dear Nurse,—You know I have for many years sent out hundreds of nurses to private cases. All these nurses are thoroughly trained and certificated, and complaints against them are uncommon. But when there are complaints, in nine cases out of ten it is not because of the nurse's ignorance or clumsiness, but because of some moral defect or unpleasant manner. Now you cannot examine a nurse in manners and morals and give her certificates for them, whereas in a home you can watch and partly judge which nurses are suitable for certain cases. For instance, a reserved woman always gets on better with men, while a cheerful, chatty nurse suits young people. I do not think the public would benefit by the register: they will pick hap-hazard some woman from the list, who may be a thoroughly bad nurse though she has passed the public examination. As for the Pension Fund, my dear child, it *is* started, and if you knew anything of business you would understand that a financial scheme backed by Messrs. Rothschild, Morgan, Gibbs, and Hambro is absolutely safe and sure of success. These are four of the best names in the City. If registration is to come, if you are then a nurse you will be allowed to be registered, whether you are a member of the British Nurses' Association or not: but you may be sure that unless a large majority of nurses wish it, there will be no registration, and at present a very small minority are in its favour. The heads of the training schools and hospital committees are also chiefly against registration.

Dear Matron,—Are you not wrong in saying a *small* miniority of nurses wish registration? Miss Wood stated at Bradford, at a meeting I attended, that over 1,000 members had already joined the Association. Should I not have a better chance of securing employment as a member of the British Nurses' Association? I do not want to leave them if there is the slightest chance of their doing good.

Dear Nurse,—A thousand *members*, yes; but I understand that on September 9th Miss Wood herself stated that there were only five or six hundred *nurses*. As for future employment, I never knew our committee ask a would-be nurse or sister whether she belonged to this or that association. Many committees, indeed, would, no doubt, regard a member of the Association as a sort of trades unionist agitator, likely to cause disaffection where peace was the rule. This danger to its members has apparently been recognised by the promoters of the Association, for lately, I believe, they have admitted lay members to their meetings in the provinces. It really cannot matter much whether you continue your subscription or not; it is a small matter to worry over, so let us turn to other things.

### ANOTHER VIEW.

I think it is necessary for the safety of a trained nurse there should be some public distinction made, guarding her against so-called nurses who are alike ignorant of sick nursing and callous of patients' feelings. It seems to me that during the last few years everyone who has "watched by a sick-bed" is called a nurse; and yet one who has borne the heavy and noble work for years within a hospital is still only classed with other nurses. Surely the British Nurses' Association is just the thing required to sift good from indifferent in the nursing world? Their object is to pass, or rather to get a bill passed, for the registration of trained nurses only.—SISTER HILDA.

### Presentation.

AT the usual monthly committee meeting of the Jenny Lind Infirmary, Norwich, held at the Institution, Pottergate Street, a pleasing event occurred in the presentation of an elegant clock to Miss Katharine Peter, the Lady Superintendent, who is about to leave for London, and whose kind and gentle manners, coupled with great ability, have been of the utmost benefit to the Institution during the term she has held office there. Miss Peter was also presented with a clock by the nurses. The committee desired to present her with that clock as a lasting and tangible proof of their confidence in her and regard for her, and they trusted the sight of it would often remind her of the Jenny Lind Infirmary at Norwich. The clock is a handsome one, which repeats the hour; and an alarum is also attached for use if desired. It is enclosed in a morocco case, and will keep time in any position. It bore the following inscription: "Presented to Miss Katharine Peter, as a parting gift from the Committee of the Jenny Lind Infirmary, Norwich, in token of regard and esteem. 1888."

### Appointments.

[It is requested that successful candidates will send a copy of their applications and testimonials, with date of election, to THE EDITOR, The Lodge, Porchester Square, W.]

QUEEN VICTORIA NURSES' INSTITUTE.—Miss Peter, late of the Royal Hospital for Sick Children, Edinburgh, has been appointed Lady Superintendent of the Scottish branch of the Queen Victoria Institute.

MONSALL FEVER HOSPITAL.—It is with great pleasure we chronicle Miss Florence Calvert's appointment as night superintendent to the above hospital. Miss Calvert was trained at Guy's, and last May we announced her appointment as sister at St. Saviour's Infirmary. Short as has been her stay at St. Saviour's, her departure is greatly regretted, and every good wish for success will follow her to her new post.

### Vacancies.

The following vacancies are announced:—

**Matrons.**

Charing Cross Hospital; £80.
Bridgewater Infirmary; £40.
Stratford-on-Avon Hospital; £40.

**Nurses.**

St. Giles' Workhouse; £20.

County of Cornwall Nurses' Home; two.

Blackheath Institute; £20.

Woolwich Garrison Female Hospital; £19.

Tunbridge Wells Hospital; £25.

Bridgewater Infirmary; £22.

Bradford Infirmary; two; £35.

St. Saviour's Infirmary, East Dulwich Grove; several; £20.

Firs Home, Bournemouth; £20.

Farm School, Redhill; £25.

Kingston Home; Hull; several.

Cheltenham Institution.

Victoria Home, Bournemouth.

Nursing Institution, 96, 97, 98*a*, Wimpole Street, London, W., Mr. Wilson, has several vacancies for thoroughly-trained Nurses.

Cancer Hospital (Free), Brompton; £25.

Nurses' Institute, Canterbury; two.

**District Nurse.**

Egham, £50.

### Notes and Queries.

*Wanted, a Hospital Guide Book.*—G. G. writes: "Is any book published containing a list of hospitals, convalescent homes, and such-like institutions? If so, will you kindly oblige myself and others with the name and publishers in the Question and Answer Column?"—"Help in Sickness," Kegan Paul, Trench, and Co., London, price 1*s*. 6*d*., will give G. G. all the information she requires in a most handy form.

**Answers.**

(69) *The Rotunda Hospital.*—If S. M. W. will apply to Dr. Macan he will give her all the particulars she requires. The fees come to about £12 for thirteen weeks.

(70) *Nursing Home.*—It depends on the amount of hospital experience. Apply to some of the homes which advertise for nurses.

(71) *The Red Cross.*—Apply to the Lady Superintendent, Netley.

